# Inferring UK COVID‐19 fatal infection trajectories from daily mortality data: Were infections already in decline before the UK lockdowns?

**DOI:** 10.1111/biom.13462

**Published:** 2021-04-07

**Authors:** Simon N. Wood

**Affiliations:** ^1^ School of Mathematics University of Edinburgh UK

**Keywords:** lockdown efficacy, lockdown impact, NPI, SARS‐CoV‐2, semiparametric

## Abstract

The number of new infections per day is a key quantity for effective epidemic management. It can be estimated relatively directly by testing of random population samples. Without such direct epidemiological measurement, other approaches are required to infer whether the number of new cases is likely to be increasing or decreasing: for example, estimating the pathogen‐effective reproduction number, *R*, using data gathered from the clinical response to the disease. For coronavirus disease 2019 (Covid‐19/SARS‐Cov‐2), such *R* estimation is heavily dependent on modelling assumptions, because the available clinical case data are opportunistic observational data subject to severe temporal confounding. Given this difficulty, it is useful to retrospectively reconstruct the time course of infections from the least compromised available data, using minimal prior assumptions. A Bayesian inverse problem approach applied to UK data on first‐wave Covid‐19 deaths and the disease duration distribution suggests that fatal infections were in decline before full UK lockdown (24 March 2020), and that fatal infections in Sweden started to decline only a day or two later. An analysis of UK data using the model of Flaxman *et al*. gives the same result under relaxation of its prior assumptions on *R*, suggesting an enhanced role for non‐pharmaceutical interventions short of full lockdown in the UK context. Similar patterns appear to have occurred in the subsequent two lockdowns.

## 1 INTRODUCTION

Clinical data on the number of cases of coronavirus disease 2019 (Covid‐19/SARS‐CoV‐2) are subject to severe temporal confounding, as the rate of testing and criteria for testing have been changing rapidly on the same time scale as the infections, particularly in the early weeks and months of the epidemic. Because these are samples of convenience where the ascertainment fraction is changing and unknown, the data can clearly not be used to infer the actual number of infections. Neither, under normal circumstances, would statisticians recommend attempting to estimate the effective reproduction number of the pathogen from such data, because given the data problems, the estimates must necessarily be driven strongly by the modelling assumptions (see, e.g. Levine *et al*., 2001, §1.6 for general discussion of the problems with inference from non‐random samples). Indeed generically, it is often very difficult to infer epidemiological parameters from clinical data, without the results being informed as much by the prior beliefs encoded in the model as by the data (e.g. Wood *et al*., 2020). Much less problematic are estimates based on randomized surveillance testing, as now conducted in the United Kingdom by the office for national statistics (see Supporting Information for discussion of inferring incidence from testing data).

However, some clinical data directly measure the quantity of epidemiological interest. This is the case for deaths with Covid‐19 and for fatal disease duration. While not perfect, these data are less compromised than the data on cases. Deaths are reliably recorded and clinical grounds for suspecting Covid‐19 are relatively clear for fatal cases, although accurately attributing death to a single cause is clearly not always possible. Good records are also often kept for such cases, with the result that there are several published studies on fatal disease duration (Verity *et al*., 2020; Linton *et al*., 2020; Wu *et al*., 2020, see Section 2). Although only possible with a delay of some weeks, it is of interest to establish what these relatively high‐quality data imply about the time course of infections, without strong modelling assumptions.

Two types of daily death data are available. Daily reported deaths (e.g. Worldometer, 2020) typically show marked weekly fluctuations as a result of weekly patterns in reporting delays, and may exclude deaths in some locations (such as nursing homes). Registered death data, such as the ONS data in the United Kingdom (Office for National Statistics, 2020), contain deaths in all locations and record exact date of death. NHS (2020) publishes equivalent data for hospital deaths in England. The weekly cycle is less pronounced in these data, but their release is necessarily delayed relative to the daily reported deaths, although recent work partially overcomes this delay problem, by modelling the delays to enable ‘now‐casting’ of deaths by actual death date: see Stoner *et al*. (2020). The right column of Figure 2 shows ONS, NHS and Swedish daily deaths by date of death (without now‐casting).

**FIGURE 1:**
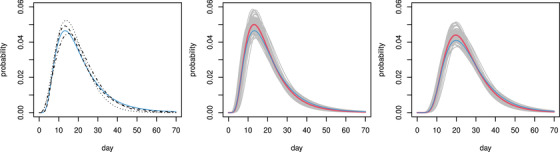
Fatal disease duration distributions. Left: onset to death. Dotted (Verity *et al*., 2020); dashed (Linton *et al*., 2020); dash‐dot (Wu *et al*., 2020); continuous blue the log‐normal mixture component for community acquired infection from the English hospital data. Middle: combined Linton–Verity–Wu onset to death model, thick red is mean model, grey are 100 draws from the distribution of the combined model, thin blue is as left. Right: as middle, but combined infection to death model. This figure appears in colour in the electronic version of this article, and any mention of colour refers to that version

**FIGURE 2:**
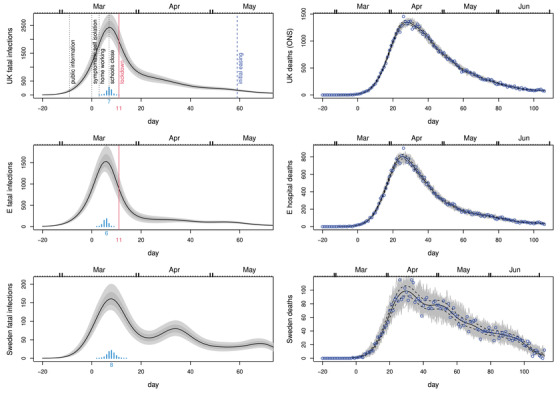
In all plots, black curves show the posterior median, while light grey and dark grey regions show, respectively, 95% and 68% confidence regions, including uncertainty in the fatal disease duration distribution. Day 0 is 13th March 2020, and the vertical red line marks the first day of UK lockdown. Top left: Inferred daily fatal infection rate, $f_{c}$, for the United Kingdom. The scaled barchart shows the posterior distribution for day of peak infection with the peak day labelled. NPI start dates are marked by labelled vertical lines. Top right: Consistency check. In grey are 100 sets of death data simulated forward from the inferred median fatal infection profile. Symbols are the ONS daily death data for the United Kingdom on which inference is based. The dashed curves are 95% confidence intervals for underlying death rate estimated by direct fitting of (1). Middle row: As top row, but using the NHS England daily hospital death data. Note that the inferred infection trajectories are substantially different from time‐lagged versions of the deaths trajectories. Bottom row: as the previous rows, but for Sweden. This figure appears in colour in the electronic version of this article, and any mention of colour refers to that version

The purpose of this paper is to show how a relatively straightforward statistical approach can be used to infer the fatal infection trajectory in the United Kingdom in a data‐driven way that makes the minimum of strong modelling assumptions. The approach is also applied to data from Sweden, the western European country offering the greatest policy contrast to the United Kingdom. Sweden never implemented full lockdown, sticking to less restrictive non‐pharmaceutical intervention (NPIs) (broadly aimed at ‘optimal mitigation’ rather than ‘suppression’ in the terms used by Walker *et al*., 2020, who projected around 40,000 deaths for this policy). Meaningful quantification of the aggregate strength of restrictions that are intrinsically multivariate is difficult, but in terms of their aggregate economic impact, Swedish GDP dropped by about 2.9% in 2020 compared to about 9.9% for the United Kingdom. Particular questions of interest are when the decline in fatal infections started in the United Kingdom and Sweden, whether UK infections were in substantial decline before full lockdown, whether the pathogen reproduction number was below 1 before lockdown, and how the timing of fatal incidence decline relates to the timing of the easing of lockdown.

Answers to these questions may contribute to judging the proportionality of lockdown measures in the UK context, where there is strong statistical evidence for very large preventable life loss being associated with economic deprivation, and of economic deprivation being increased by economic shocks. This evidence is reviewed in detail in Marmot *et al*. (2020). For example, the deprivation‐related life loss that the current UK population was due to suffer before the Covid crisis was 140–240 million life years (or 2–3.5 years per capita, see Marmot *et al*., 2020, figure 2.3, for example). The range depends on whether the life expectancy of the lower decile or the lower half of the deprivation distribution is used as the reference for achievable life‐expectancy. In examining the effects of the 2008 financial crisis and its aftermath, Marmot documents sharp reductions in life expectancy growth in the United Kingdom, which would imply a life loss burden in the 10s of millions of years. However, attribution of such reduction‐relative‐to‐trend is obviously very difficult. Less problematic is the 9 million life year loss implied by the increase in life expectancy gap between the more and less deprived halves of the UK population since 2008 (7 weeks per capita, see Marmot *et al*., 2020, figure 2.5, for example): given the evidence presented in the review, this is more difficult to attribute to causes unrelated to the 2008 economic shock. The Bank of England estimates the shock to the UK economy caused by the response to Covid‐19 to have been the largest for over 300 years, so there is a clear danger of substantial life loss being caused, given the historical data for the United Kingdom. For example, a feature of the 2008 crisis already repeated in 2020 is the reliance on a large programme of quantitative easing. Quantitative easing is credibly argued to directly increase economic inequality via mechanisms related to asset price inflation (e.g., Domanski *et al*., 2016; Fontan *et al*., 2016). There is some literature attributing some short‐term life‐saving to recessions, see, for example, Anon (2020), but the effects are modest relative to the long‐term effects reviewed by Marmot. For comparison with the above figures, the life loss that might have occurred from a minimally mitigated Covid‐19 epidemic appears to be in the region of 3 million life years (2.5 weeks per capita). This is based on Office for National Statistics (2019) lifetables, ONS Covid‐19 fatality by age data, a mid‐range infection fatality rate estimate of 0.006, a somewhat high herd immunity threshold of 0.7 and a 1‐year lower bound life expectancy adjustment for co‐morbidities based on Hanlon *et al*. (2020). It is broadly in line with the UK government estimates (Anon, 2020). Given that 9 million life years, associated with the substantially smaller economic shock of 2008, is not negligible relative to 3 million life years potentially losable to Covid‐19, there is obviously a delicate balance to be struck in the UK context, and evidence based on assumption light inference should probably play a role in shaping that balance. Another indicator of the difficulty of achieving the right balance is that the usual UK threshold for approving a pharmaceutical intervention is £30,000 per life year saved. On the basis of economic costs detailed in OBR (2020) and the preceding life loss figures, the NPIs used in the United Kingdom appear to have a cost per life year saved that is an order of magnitude higher than this (excess government borrowing is projected to peak at £660 billion in the OBR central scenario, for example). This discrepancy in willingness to pay may lead to a problem of opportunity cost, as the same money cannot be spent on preventing other life loss, such as that associated with economic hardship.

The remainder of the paper is structured as follows. Section 2 discusses the available information on the distribution of fatal disease durations, and how to combine it while adequately characterizing the associated uncertainty. Section 3 introduces a simple generalized additive model for direct modelling of the daily deaths trajectories, and shows how it can be extended to infer the trajectory of fatal infections, either directly or by inferring the trajectory of the pathogen effective reproduction number, *R*, in a simple epidemic model. Since the extensions are not standard models, and are relatively expensive to compute with using standard Bayesian software, Section 4 outlines methods allowing computationally efficient inference with the models. Section 5 presents the main results on infection trajectories, and also the estimation of *R*. Section 6 discusses possible problems with the approach, in particular examining whether smoothness assumptions could be leading to substantial bias in inferred timings. Replication code and data are provided in the Supporting Information.

## 2 FATAL DISEASE DURATION

Data on the incubation period from infection to onset of symptoms are analysed in many papers, for example, Lauer *et al*. (2020) found that the period is 2–11 days for 95% of people, with a median of 5.2 days. A meta‐analysis by McAloon *et al*. (2020) suggests a log‐normal distribution with log scale mean and standard deviation of 1.63 and 0.50. The uncertainty in this distribution is negligible in comparison to the uncertainty in the distribution of times from onset of symptoms to death discussed next.

Several studies estimate the distribution of time from onset of symptoms to death, while properly controlling for the right truncation in the fatal duration data. Verity *et al*. (2020) found that the distribution of time from onset of symptoms to death for fatal cases can be modelled by a gamma density with mean 17.8 and standard deviation 8.44, based on 24 patients from Wuhan. Wu *et al*. (2020) suggested a gamma density model with mean 20 and standard deviation 10 based on 41 patients from Wuhan. Linton *et al*. (2020) found that a log normal model offers a slightly better fit, and estimated a mean of 20.2 days and standard deviation of 11.6 days from 34 patients internationally. These distributions are shown in the left panel of Figure 1. A simple meta‐analysis approach was used to combine the models. Samples of the correct size were simulated from each model and a log normal model was estimated by maximum likelihood for the combined resulting sample (n=99). A further log normal was also fitted (minimizing Kullback Leibler divergence) to the infection to death distribution implied by the fitted onset to death distribution and McAloon *et al*. (2020) infection to onset distribution (treated as independent). This process was repeated to generate replicate distributions. These replicate distributions were treated as draws from the distribution of infection to death distributions in subsequent analysis. One hundred such draws are shown in Figure 1. The log normal was chosen because the careful analysis of Linton *et al*. (2020) found it to be a better model than the gamma.

In addition, under strict conditions, I was able to access data on fatal disease durations for deaths occurring in English hospitals. Access to data with hospital acquired infections filtered out was not possible, so is was necessary to treat these data as a mixture of hospital‐ and community‐acquired infections, as detailed in the Supporting Information. The resulting inferred fatal disease duration distribution for community acquired infection is plotted in blue in Figure 1 (this figure appears in colour in the electronic version of this article, and any mention of colour refers to that version), and is consistent with the published studies.

## 3 MODELS

This section first introduces a simple generalized additive model for daily death trajectories, and then shows how this can be extended to directly infer the trajectory of fatal infections (fatal incidence), without having to assume any particular dynamic model for the epidemic. The resulting model is no longer a generalized additive model and is the model that this paper advocates using. Its structure is such that any method for inference with the model can also be used for inference with the dynamic model of Flaxman *et al*. (2020), with appropriate restriction of the incidence trajectory to one representable with that model. The Flaxman model is presented to allow comparison of the results from the infection trajectory model with the apparently contradictory results of Flaxman *et al*., but not to advocate its use.

### 3.1 Basic deaths series model

Let $y_{i}$ denote the deaths or reported deaths on day *i*, assumed to follow a negative binomial distribution with mean $\mu_{i}$ and variance $\mu_{i}+\mu_{i}^{2}/\theta$. Let

(1) $$\log\left(\mu_{i}\right)=f\left(i\right)+f_{w}\left(D_{i}\right),$$

where *f* is a smooth function of time measured in days, and $f_{w}$ is a zero mean cyclic smooth function of day of the week, $D_{i}\in \left\{1,2,\ldots ,7\right\}$, set up so that $f_{w}^{\left[k\right]}\left(0\right)=f_{w}^{\left[k\right]}\left(7\right)$, where k=0,1, or 2 denotes order of derivative. f(i) represents the underlying log death rate, whereas $f_{w}$ describes the weekly variation about that rate. The functions *f* and $f_{w}$ can be represented using splines with associated smoothing penalties $\lambda \int f^{\prime \prime }{\left(t\right)}^{2}dt$ and $\lambda_{w}\int f_{w}^{\prime \prime }{\left(D\right)}^{2}dD$. Hyper‐parameters λ and $\lambda_{w}$ control the smoothness of the functions. The model is a straightforward generalized additive model and $\left(\lambda ,\lambda_{w}\right)$ can be estimated as part of model fitting using a standard empirical Bayes approach as described in Wood (2017). The model provides a good fit to both the reported deaths and ONS data. As expected $f_{w}$ is greatly attenuated for the ONS data (it vanishes for Swedish exact death date data).

### 3.2 Infection trajectory model

To estimate the daily infection trajectory, the model is extended by expressing f(i) in terms of the time course of earlier infections. Let $f_{c}\left(i\right)$ be the function describing the variation in the number of eventually fatal infections over time. Let B be the square matrix such that $B_{ij}\;=\;\pi \left(i\;-\;j\;+\;1;\mu ,\sigma^{2}\right)$ if i≥j and 0 otherwise. π denotes an infection‐to‐death log normal density as discussed above. For the moment, its parameters, μ and σ^2^, are treated as fixed but this will be relaxed in Section 4.3. Given the continuity of the log normal, the given form for $B_{ij}$ can be viewed as approximating an integral of π over each day, using the midpoint of the integrand – it is straightforward to approximate the integral more accurately, but given that π is originally estimated from durations discretized to whole days, any precision gain is illusory. If $f_{c}={\left[f_{c}\left(0\right),f_{c}\left(1\right),\ldots \right]}^{T}$ and $\delta ={\left[\delta \left(1\right),\delta \left(2\right),\ldots \right]}^{T}$, then $\delta ={\mathrm{Bf}}_{c}$, where δ(i) is the expected number of deaths on day *i*. $\log f_{c}\left(i\right)$ can be represented using a spline basis, again with a cubic spline penalty. Working on the log scale ensures that $f_{c}$ is positive, but is also appealing because it means that a cubic spline penalty on $\log f_{c}\left(i\right)$ can be interpreted as a first derivative penalty $\int r^{\prime }{\left(t\right)}^{2}dt$, acting on the epidemiologists ‘intrinsic growth rate’, *r*. The final infection trajectory model is then obtained by simply substituting f(i)=logδ(i) into (1). B is rank deficient, so inferring $f_{c}$ can be viewed as an inverse problem: without regularization, multiple solutions that oscillate from day‐to‐day are possible. This ambiguity is removed by the smoothing penalty on $\log f_{c}$.

### 3.3 Relaxed Flaxman model

In the time since this work was originally undertaken in late April 2020, the work of Flaxman *et al*. (2020) has appeared. Flaxman *et al*. make inferences about the reproduction number, *R*, and hence incidence rates, based on death trajectories and the fatal infection duration distribution of Verity *et al*. (2020), but do so by modelling the pathogen‐effective reproduction number $R_{t}$ within a simple epidemic ‘renewal model’. Flaxman *et al*. (2020) represent $R_{t}$ as a step function with steps allowed each time the government announced new containment interventions, and a sparsity prior promoting a small number of steps. In the notation of Flaxman *et al*., the expected number of infections each day (now total, rather than fatal) are denoted as $c_{t}$. Given an initial *c*_1_, the model is iterated from t=2 as follows:

(2) $$c_{t}=\left(1-\sum_{i=1}^{t-1}c_{i}/N\right)R_{t}\sum_{\tau =1}^{t-1}c_{\tau }g_{t-\tau }$$

where *N* is the total initially susceptible population, $g_{1}=\int_{0}^{1.5}\gamma \left(x\right)dx$ and $g_{j}=\int_{j-.5}^{j+.5}\gamma \left(x\right)dx$ for j>1. γ is the p.d.f. of a Gamma distribution with shape parameter 6.5 × 0.62^2^ and scale parameter 0.62^−2^. The $c_{t}$ values multiplied by the assumed infection fatality rate give $f_{c}$. The level of the infection fatality rate (IFR) only matters for the damping term in the first bracket of the expression for $c_{t}$ – this has almost no effect in practice, a mid‐range value of 0.006 was used. The original assumptions about $R_{t}$ can be relaxed by representing $\log R_{t}$ using a spline basis, with associated penalty as for the other models, while $\log c_{1}$ is also treated as a free parameter. Hence, $f_{c}$ in the infection trajectory model can simply be replaced by the Flaxman model with $\log R_{t}$ represented as a spline function. The model is otherwise unchanged. This model is presented only to allow comparison of this paper's results with those of Flaxman *et al*. (2020): its simple single‐compartment structure clearly does not meet the aim of inferring incidence with minimal assumptions.

## 4 METHODS

The infection trajectory and Flaxman renewal models are not standard models estimable with standard software. They can be implemented in Bayesian software, such as JAGS or STAN, but inference typically takes several hours if this is done. Dealing adequately with the uncertainty in the disease duration distribution multiplies this cost by one to two orders of magnitude. To avoid these problems, an empirical Bayes approach can be employed.

### 4.1 Basic inferential framework

Direct inference about (1) uses the empirical Bayes approach of Wood *et al*. (2016) in which the smooth functions are estimated by penalized likelihood maximisation (e.g. Green and Silverman, 1994), with the smoothing parameters and θ estimated by Laplace approximate marginal likelihood maximization. Writing β for the combined vector of basis coefficients for *f* and $f_{w}$, the penalized version of the log likelihood, l(β), can be written

$$\begin{array}{ccc} & & l\left(\beta \right)-\frac{\lambda }{2}\int f^{\left[2\right]}{\left(t\right)}^{2}dt-\frac{\lambda_{w}}{2}\int f_{w}^{\left[2\right]}{\left(D\right)}^{2}dD\\ & & \;=\;l\left(\beta \right)-\frac{1}{2}\beta^{T}S_{\lambda }\beta , \end{array}$$

where $S_{\lambda }=\lambda S_{f}+\lambda_{w}S_{w}$: $S_{f}$ and $S_{w}$ are known constant positive semi‐definite matrices. Smoothing parameters, λ and $\lambda_{w}$, control the smoothness of *f* and $f_{w}$. Let $\hat{\beta }$ be the maximizer of the penalized log likelihood, and H its negative Hessian at $\hat{\beta }$. Viewing the penalty as being induced by an improper Gaussian prior, $\beta ∼N(0,S_{\lambda }^{-})$, $\hat{\beta }$ is also the maximum a posteriori (MAP) estimate of β. Furthermore, in the large sample limit,

(3) $$\beta |y∼N(\hat{\beta },{\left(H+S_{\lambda }\right)}^{-1}).$$

Writing the density in (3) as $\pi_{g}$, and the joint density of **y** and β as π(y,β), the Laplace approximation to the marginal likelihood for the smoothing parameters λ and θ is $\pi \left(\lambda ,\theta \right)=\pi \left(y,\beta \right)/\pi_{g}\left(\beta |y\right)$. Nested Newton iterations are used to find the values of log(λ),θ maximizing π(λ,θ) and the corresponding $\hat{\beta }$ (for details, see Wood *et al*., 2016).

Given (3), credible intervals for *f* are readily computed, but it is also straightforward to make inferences about when the peak in *f* occurs. Simply simulate replicate coefficient vectors from (3) and find the day of occurrence of the peak for each corresponding underlying death rate function, *f*.

### 4.2 Extension for the infection and Flaxman models

Although inference about (1) using the preceding framework requires little more than a call to the gam function in R package mgcv, its application to the other models, which are not generalized additive models, requires more work. For the model formulated in terms of $f_{c}$, this requires expressions for the negative binomial deviance (or log likelihood) and its derivative vector and Hessian matrix w.r.t. the model coefficients.

First, consider the negative binomial deviance for observation *i*,

$$\begin{array}{ccc} D_{i} & = & 2y_{i}\log\left\{\max\left(1,y_{i}\right)/\mu_{i}\right\}\\ & & -\;\left(y_{i}+\theta \right)\log\left\{\left(y_{i}+\theta \right)/\left(\mu_{i}+\theta \right)\right\}, \end{array}$$

$$\begin{array}{ccc} \frac{dD_{i}}{d\mu_{i}} & = & 2\left(\frac{y_{i}+\theta }{\mu_{i}+\theta }-\frac{y_{i}}{\mu_{i}}\right)\;\text{and}\\ \frac{d^{2}D_{i}}{d\mu_{i}^{2}} & = & 2\left(\frac{y_{i}}{\mu_{i}^{2}}-\frac{y_{i}+\theta }{{\left(\mu_{i}+\theta \right)}^{2}}\right). \end{array}$$

These need to be transformed into derivatives w.r.t. β, as follows:

$$\begin{array}{ccc} \frac{\partial D_{i}}{\partial \beta_{j}} & = & \frac{dD_{i}}{d\mu_{i}}\frac{\partial \mu_{i}}{\partial \beta_{j}}\;\text{and}\\ \frac{\partial^{2}D_{i}}{\partial \beta_{j}\partial \beta_{k}} & = & \frac{d^{2}D_{i}}{d\mu_{i}^{2}}\frac{\partial \mu_{i}}{\partial \beta_{j}}\frac{\partial \mu_{i}}{\partial \beta_{k}}+\frac{dD_{i}}{d\mu_{i}}\frac{\partial^{2}\mu_{i}}{\partial \beta_{j}\partial \beta_{k}}. \end{array}$$

Writing $X^{f}$ and $X^{w}$ for the model matrices for the smooth terms $\log f_{c}$ and $f_{w}$, we have $\delta ={\mathrm{Bf}}_{c}$ where $f_{c}=\exp\left(X^{f}\beta^{f}\right)$ (here exp(·), division and multiplication are applied element‐wise to vectors), and $f_{w}=X^{w}\beta^{w}$. Then $\mu =\exp(\log\delta +f_{w})$, whereas

$$\frac{\partial \mu^{\;}}{\partial \beta^{f}}=\text{diag}\left(\mu /\delta \right)B\frac{\partial f_{c}}{\partial \beta^{f}},\;\;\;\frac{\partial \mu^{\;}}{\partial \beta^{w}}=\text{diag}\left(\mu \right)X^{w},$$

$$\frac{\partial^{2}\mu }{\partial \beta_{j}^{w}\partial \beta_{k}^{w}}=\mu X_{\cdot ,j}^{w}X_{\cdot ,k}^{w},\;\;\;\frac{\partial^{2}\mu }{\partial \beta_{j}^{f}\partial \beta_{k}^{f}}=\text{diag}\left(\mu /\delta \right)B\frac{\partial^{2}f_{c}}{\partial \beta_{j}^{f}\partial \beta_{k}^{f}}$$

$$\;\text{and}\;\frac{\partial^{2}\mu }{\partial \beta_{j}^{f}\partial \beta_{k}^{w}}=\text{diag}\left(X_{\cdot ,k}^{w}\mu /\delta \right)B\frac{\partial f_{c}}{\partial \beta^{f}}.$$

For the given representation of $f_{c}$

$$\frac{\partial f_{c}}{\partial \beta^{f}}=\text{diag}\left(f_{c}\right)X^{f}\;\;\text{and}\;\;\frac{\partial^{2}f_{c}}{\partial \beta_{j}^{f}\partial \beta_{k}^{f}}=\text{diag}\left(f_{c}\right)X_{\cdot ,j}^{f}X_{\cdot ,k}^{f}.$$

When using the relaxed Flaxman model, the preceding derivatives of $f_{c}$ have to be replaced with derivatives of $f_{c}$ w.r.t. the coefficients of the spline representing $\log R_{t}$. Routine application of the chain rule to (2) gives corresponding iterations for the derivatives of $c_{t}$, and hence $f_{c}$, w.r.t these spline coefficients and $\log c_{1}$.

Given these expressions and the penalties, $\hat{\beta }$ can be obtained by Newton iteration, given smoothing parameters. To estimate smoothing parameters, the simplest approach is to fix the negative binomial θ at its estimate from model (1), and use Wood and Fasiolo (2017), alternating generalized Fellner Schall updates of the smoothing parameters with updates of $\hat{\beta }$ given those smoothing parameters. This finds the smoothing parameters to approximately maximize the model marginal likelihood. The non‐linearity of the renewal equation model means that some effort is required to get non‐absurd starting values. I got these by a few minutes of experimentation with simple step functions for the initial $\log R_{t}$ to get death trajectories of roughly the shape and amplitude of the true trajectories (a close initial fit is not required: initial deviances two orders of magnitude greater than for the final fit were unproblematic).

Given θ and the smoothing parameters, the approximate posterior (3) could be used directly, or as the basis for the proposal distribution in a simple Metropolis Hastings sampler. A fairly efficient sampler results from alternating fixed proposals based on (3) with random walk proposals based on zero mean Gaussian steps with a shrunken version of the posterior covariance matrix. By this method, effective sample sizes >5000 for each coefficient took about 40 s computing on a low specification laptop. This was the approach used for the infection trajectory model. The results were indistinguishable from those produced at the cost of several hours of computing using JAGS (Plummer, 2003; Plummer *et al*., 2006) to simulate from the model posterior.

### 4.3 Disease duration distribution uncertainty

The methods so far perform inference conditional on fixed values for the parameters μ and σ^2^ of the log normal density describing the infection to death duration distribution. In reality, there is uncertainty about these parameters. To incorporate this uncertainty into the infection trajectory model, inference was run for each of the 100 sample distributions shown in grey in the right‐hand panel of Figure 1, and the resulting posterior samples were pooled, to give a sample from the unconditional posterior distribution of the model.

## 5 RESULTS

Figure 2 shows the results of applying the model to the Office for National Statistics daily Covid‐19 death data for the United Kingdom, to the NHS England hospital data and to the daily death data for Sweden from Folkhälsomyndigheten (2020). The results include the uncertainty about the disease duration distribution shape. ONS and NHS data are up to 27th June – including later data simply narrows the uncertainty, while making negligible difference to the overall conclusions. The most notable feature of the results is that fatal infections are inferred to be in substantial decline before full lockdown (the same result was obtained by this method in early May 2020, based on the first 50 days of reported daily death data). Sweden appears most likely to have peaked only 1 or 2 days later (barring some systematic difference in fatal disease durations for Sweden), having introduced NPIs well short of full lockdown. The results also emphasize the fact that the infection trajectory is not simply a time‐shifted version of the death trajectory (assuming that it was, might lead to unwarranted delay in easing lockdown, for example). The difference in timing and shape of the inferred profile between the ONS and NHS data reflects the fact that the latter contain care home data. There is an argument for preferring hospital data for inferring community fatal infections, in that the care home epidemic is now known to have special features with at least some of the infection not coming from normal community transmission. See, in particular, Comas‐Herrera *et al*. (2020) for a discussion of care home deaths internationally, including the United Kingdom. In addition, in the United Kingdom, care home deaths were often attributed to Covid‐19 without a test, especially after death certification guidelines were changed to encourage reporting of suspected, rather than confirmed, Covid‐19 deaths. The care home data therefore have some under‐reporting of Covid deaths, followed by over‐reporting (the signal of this is visible in ONS data in the change in non‐Covid pneumonia deaths being reported, relative to normal, for example).

**FIGURE 3:**
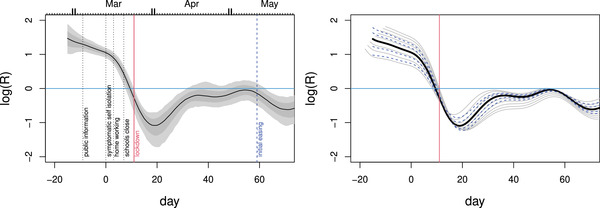
Left: Estimates and confidence bands for the effective reproduction number, *R*, from a simple SEIR model given the inferred infection profile (incidence), $f_{c}$. The assumed mean time to infectivity was 1/γ=3 days and the mean infectivity duration was 1/δ=5 days. The labelled vertical bars show policy change dates in March 2020. Given the rapidity of policy change relative to the epidemic's dynamic time scale, and government policy sometimes lagging behaviour, casual over interpretation of these timings should be avoided. Right: sensitivity analysis. Dashed blue: time to infectivity was varied from 1 to 5 days. Grey:‐ duration of infectivity was varied from 2 to 10 days. Logs are natural. *R* appears to be below 1 before full lockdown, but fell further after it. This figure appears in colour in the electronic version of this article, and any mention of colour refers to that version

Taken together the results for the United Kingdom and Sweden raise the questions of firstly whether full lockdown was necessary to bring infections under control, or whether more limited measures might have been effective, and secondly whether the several month duration of full lockdown was appropriate. These emphasize the desirability of statistically well‐founded direct measurement of epidemic size through randomized testing. Had such testing being carried out leading up to lockdown it would have been clearer if the measures preceding lockdown (see Figures 2 and 3) were working, or whether stronger restrictions were needed. Similarly, such testing might have given earlier indication of when lockdown could be eased. Instead, management was reliant on a complex modeling synthesis of expert judgement and problematic clinical case data. Less statistically problematic reconstructions, like the one presented here, are clearly only possible weeks after the fact. Note that although it is natural to interpret these fatal infection trajectories as proportional to the overall infection trajectories, that will only be the case if the infection fatality rate is constant over time. There is evidence for improvements in hospital care from late March onwards that suggest that this is might not be the case (see Dennis *et al*., 2021). The Supporting Information includes a sensitivity analysis of this issue: it has the potential to right shift the peak incidence by up to a day and to lead to somewhat less rapid decay of the incidence trajectory.

### 5.1 Inferring R

Much public debate has focused on the effective reproduction number, *R*, and in theory, it is possible for a decline in the rate of infections to be only temporary as a result of *R* dropping but remaining above one. Could it be that the declines in $f_{c}$ seen before lockdown were of this short‐term type, and that renewed increase would therefore have occurred without full lockdown? The answer appears to be no. *R* is all but impossible to measure directly, so inference about it requires assumption of an epidemic model. However, given an epidemic model, it can be directly inferred from the reconstructed infection profile. For example, consider a simple susceptible exposed infectious recovered (SEIR) model: $\dot{S}=-\beta SI$, $\dot{E}=\beta SI-\gamma E$, $\dot{I}=\gamma E-\delta I$ (here δI is the rate of recovery *or* progression to serious disease).${\hat{f}}_{c}$ is a direct estimate of βSI (to within a constant of proportionality), so by solving

$$\dot{E}={\hat{f}}_{c}-\gamma E,\;\;\;\;\;\;\dot{I}=\gamma E-\delta I$$

(from 0 initial conditions) the direct estimate $R=f_{c}/\left(I\delta \right)$ is readily computed (any constant of proportionality cancels in *R*). A different epidemic model could be used here of course: see Diekmann *et al*. (1990) for calculation of *R* in general from a model. Figure 3 shows the results using ${\hat{f}}_{c}$ for the English hospital data for plausible values of average time to infectivity of 1/γ=3 days and mean duration of infectiousness of 1/δ=5 days, along with sensitivity analysis for these values. The credible intervals shown include the uncertainty about the fatal disease duration distribution. *R* appears to be below 1 before full lockdown.

A useful feature of the *R* estimates is to emphasize that the analysis in this paper in no way suggests that lockdown did not have an effect on transmission. Even if *R* was below 1 before lockdown, full lockdown can only have reduced it further, and the estimates in Figure 3 are obviously consistent with this. Note, however, that the recovery in *R* after the post‐lockdown dip is to be expected, given the simple fact that *R* is the number of new infections created per infection, *averaged over the population of infections*, not the population of people. Broadly speaking, at lockdown, the population of people, and infections, was split into the locked down population, where infections could create few new infections, and the ‘unlocked’ population where the reproductive rate of the pathogen was higher (assuming lockdown had an effect). An initial average over all infections is then dominated by those infections in the locked down population, giving a low *R* (especially once the possibilities for infecting locked down household members have been exhausted). As the infections in the locked down population die out, the proportion of all infections that are in the unlocked population must increase – so that an average over all infections must yield a higher *R* again.

### 5.2 The Flaxman model

As noted above in Section 3, Flaxman *et al*. (2020) also analysed death trajectories, using a simple epidemic model, but came to conclusions apparently contradicting Figure 3. They concluded that only after full lockdown did *R* drop below 1, and that fatal infections continued to increase up until the eve of full lockdown. Flaxman *et al*. (2020) used the Verity *et al*. (2020) fatal disease duration distribution, so the difference in results does not lie there. To describe the epidemic dynamics, Flaxman *et al*. use the simple single compartment discrete renewal model (2). Within that model, they assume that *R* is constant between the imposition of interventions, but can undergo a step change at each intervention: the steps are free model parameters. This model for *R* is quite restrictive. In particular, it does not allow *R* to change after lockdown, despite the fact that at lockdown, the population has been stratified in a way that the renewal model does not represent, so that some compensating flexibility in *R* is likely to be required to avoid modelling artefacts. At the same time, the model is rather underdetermined preceding lockdown, because of the frequent intervention changes. This indeterminacy in the model is addressed by using a sparsity promoting prior on the step changes in *R*, which favours few larger changes, rather than several smaller changes (see the supplementary material for Flaxman *et al*. for a description of this prior). When using the model to simultaneously model multiple European countries, there is a further assumption that the intervention effects are the same for all countries (despite the different order of their implementation) and that only the lockdown effect varies between countries. It seems likely to be difficult to pick up effects of the interventions preceding lockdown from such a model structure.

**FIGURE 4:**
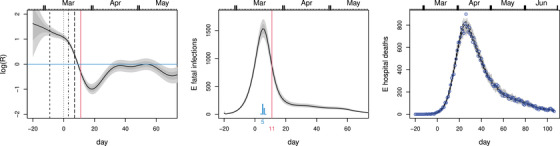
Results from the epidemic model of Flaxman *et al*. (2020), with the assumptions on *R* relaxed: logR is assumed smooth and continuous. Left: the inferred *R* from fitting the NHS hospital data. The inferred *R* trajectory is similar to the one shown in Figure 3, despite the different model structure. Intervals do not include disease duration distribution uncertainty here. Middle: the corresponding fatal infection profile. Right: the simple sanity check as in Figure 2. This figure appears in colour in the electronic version of this article, and any mention of colour refers to that version

A relaxed version of the Flaxman model in which $\log R_{t}$ is a continuous function is described in Section 3. The results from using this model for inference using the NHS hospital data are shown in Figure 4. The relaxation of the assumptions on *R* brings the results (for the United Kingdom) into alignment with those in the rest of this paper, and into broad consistency with developments later in the year, which are otherwise difficult to square with Flaxman *et al*. (2020).

### 5.3 Later infection waves

Although the initial motivation for this work was to provide reasonably timely analysis for the first wave, based on the limited data available by May 2020, the methods scale readily to the much longer data runs available by early 2021. The only change is that it makes sense to use an adaptive smoother (see, e.g. Wood, 2017, §5.3.5) for f(t), in which the degree of smoothness is allowed to vary with time. The longer data runs make it feasible to estimate the multiple smoothing parameters that this entails. Using an adaptive smooth guards against artefacts driven by the smoothness that is appropriate on average, for all the data, not being appropriate at times of rapid change.

**FIGURE 5:**
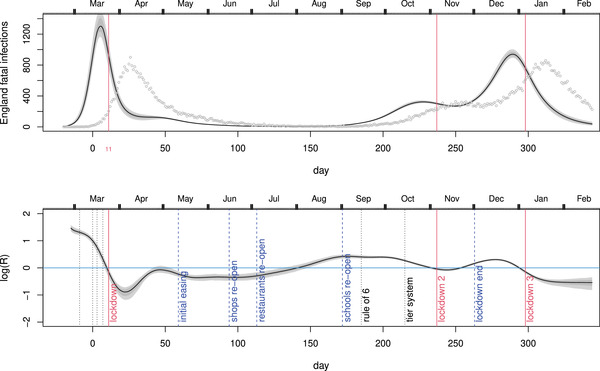
Inference for the English hospital deaths data up to mid‐February 2021, including disease duration uncertainty. Top: inferred fatal incidence. Grey symbols are the hospital deaths from which incidence is inferred. Red vertical lines mark the start of each of the three English lockdowns. Note that improvements in medical treatment mean that the IFR is very likely not to be constant between the first and later waves, so interpreting their relative sizes in terms of total infections is difficult. Bottom: the inferred *R* using the simple SEIR approach. NPI impositions short of lockdown are marked by dotted vertical lines, and relaxations are marked by dashed lines. This figure appears in colour in the electronic version of this article, and any mention of colour refers to that version

The results of this application are shown in Figure 5. Note that likely changes in infection fatality rate as a result of improved hospital treatment mean that the relative sizes of the fatal infection incidence curves in the first and subsequent waves cannot be interpreted as reflecting the relative sizes of total incidence (the later incidence curves would need to be scaled up somewhat). Causal over‐interpretation of the *R* curves should be avoided, not least because there is no reason to expect Covid‐19 not to display the seasonality in transmission common to other respiratory illnesses. However, the results are obviously inconsistent with full lockdowns having caused R<1, because cause should not happen after effect. Further, the drop in *R* seen after the initial NPIs were introduced, but before full lockdown, does seem consistent with the levels of *R* later achieved while measures short of lockdown were in place. The interesting feature of *R* apparently increasing from quite early in the second lockdown might relate to the spread of the new variant, but, of course, also occurs at a time when respiratory infections generally start to increase. Likewise, the further increase until mid‐December may well be due to the new variant, but increased activity in the run up to Christmas is also likely to be a factor – incidence appears to peak over the Christmas to New Year period. Vaccine rollout seems virtually certain to be a major factor in pushing down *R* and fatal incidence from December. The vaccine has been given to those most at risk first, so the constant IFR assumption required to interpret fatal incidence as proportional to total incidence obviously no longer holds. This further implies that the inferred *R* is in some sense only the *R* relevant to the ‘at serious risk’ population. Of course, it could be argued that for epidemic management purposes, the fatal incidence and the corresponding *R* are of primary interest.

Interestingly, the pattern observed at the second lockdown and in the preceding months is consistent with the results reported by Knock *et al*. (2020) who analysed regionally stratified death, hospital occupancy and testing data for 2020 up until December, using a highly detailed age structured SEIR with added health service compartments. The entire trajectory up until December is also consistent with the results of Wood and Wit (2021), who reimplemented the Knock *et al*. model, but removed some of its very strong modelling assumptions around the first lockdown.

## 6 MODEL CHECKING

Although standard residual checks indicate no problem with the model from the point of view of statistical fit, there are three issues which could potentially undermine the results, and a further issue relating to interpretation.

The first relates to the infection to death interval distribution and the fact that the death data contain an unknown proportion of patients whose infection was hospital acquired. These patients are likely to have had shorter disease durations, because they were already sufficiently unwell or frail to be in hospital. This paper has inferred when the fatal infections would have occurred if they were all community generated, because it is the community infections that are of interest with respect to the effects of lockdown, social distancing, and so on. Without knowing even the proportion of deaths from hospital acquired infection, it is anyway not possible to do otherwise.

The presence of hospital infections in the death data will bias inference about the dynamics of community fatal infections if it substantially changes the shape of the deaths profile, relative to what would have occurred without hospital infection. Broadly, if the trajectory of hospital acquired infection deaths peaked earlier than the overall trajectory, then the community infection peak will be estimated to be earlier than it should be (because the true community infection death peak is then later). Conversely, if the hospital acquired infection deaths peaked later, then the community infection peak will be estimated as being later than it should be. The degree of bias will depend on the proportion of hospital acquired infections and the degree of mismatch in timings. It is difficult to judge which alternative is more likely: standard epidemiological modelling assumptions would imply that the more community acquired cases are hospitalized the more hospital infections would occur and that hospital infections will lag community cases. But against this, hospital acquired fatal disease durations are likely to contain a higher proportion of shorter durations. In any case, the proportion of hospital acquired infections in the death series would have to be quite high for the issue to substantially modify the conclusions.

The second issue is that age dependency in the duration distribution coupled with shifts in the age structure of deaths over time could also be problematic. However, as documented in the Supporting Information, the data for England and Wales show remarkably little variation in the age structure of Covid‐19 fatalities over the course of 2020, whereas analysis of English hospital data apparently shows little evidence for age dependence in the disease duration distribution.

The third issue is whether the smoothing penalty on $\log f_{c}$ would lead to systematic mis‐timing of the estimated peak under the scenario of a very asymmetric peak in the true infection profile around lockdown. To investigate this, data were simulated from a model in which the underlying infection rate increased geometrically, doubling every 3 days until lockdown, when the rate dropped immediately to 0.2 of its peak value, shrinking thereafter by 5% per day. Fatal infections were simulated as Poisson deviates with the given underlying rate. This model is an extreme scenario, in which measures prior to full lockdown had no effect, and the effect of lockdown was instant, as if the locked down population (i.e. those not in essential work) had isolated alone, rather than increasing their contact with members of their household while drastically reducing it with everyone else. However, it is the scenario implicit in much public discussion in the United Kingdom, at least at the time that this work was originally conducted. Under this scenario, the method does indeed tend to incorrectly estimate the infection peak as 2–3 days before lockdown, rather than the day before, as it struggles to accommodate the drop.

The naive approach to this issue is to introduce a parameter at lockdown representing an instantaneous drop in infections. However, doing so introduces a very strong structural assumption into the model, undermining the aim of avoiding strong assumptions. This approach also has the serious side effect of introducing non‐parametric smoothing boundary effects on both sides of the break. These boundary effects severely compromise inference in the most interesting region of the infection profile, while simultaneously increasing the importance of the structural assumption at the expense of the data. Indeed, when such a model is built, it estimates a large drop even from data simulated from a smooth infection profile. It also estimates such a drop if the drop's location is moved (for simulated or real data).

A better approach is to use a smooth time dilation to relax, but not eliminate the model smoothness assumptions in the vicinity of lockdown. The dilation is made sufficient that the model can accurately capture the extreme scenario in the simulation, but without imposing a break and boundary effects. In particular, $f_{c}$ and its smoothing penalty are computed with respect to a version of time which makes the day before, of and after lockdown count as 3.5, 6 and 3.5 days, respectively. Obviously, regular un‐dilated time is used for mapping infections to deaths. For the extreme simulation, the model then correctly gives most posterior probability to the day before lockdown as the peak. In contrast, the same model for the real data has very low probability of the peak being the day before lockdown rather than earlier.

**FIGURE 6:**
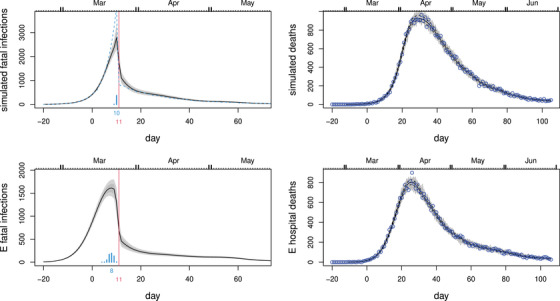
Model checking plots in which the smoothness assumptions are relaxed around lockdown by a time dilation, in order to allow accurate capture of any extremely discontinuous infection profile in this region. The top row shows the method reconstructing an extreme simulation scenario in which there was no reduction in transmission rate up until lockdown, and then an instantaneous drop. Left: the reconstruction (plot meaning as Figure 2) with the true simulated daily infections shown dashed. Right: forward simulation from the median profile as in Figure 2. The blue symbols are the simulated death data used for inference. The bottom row is for the NHS England hospital data under the time‐dilated model. Even this model deliberately modified to promote a very abrupt change at lockdown suggests that the infection rate was probably declining before lockdown. This figure appears in colour in the electronic version of this article, and any mention of colour refers to that version

Figure 6 shows the results from fitting the time‐dilated model to the extreme simulation scenario and to the NHS England hospital data. Even this model, deliberately modified to favour a very abrupt change at lockdown, suggests that infections started to decline before lockdown, with the most likely day for the peak only 1 day later than with the un‐dilated model. The Supporting Information includes similar checks for the Flaxman *et al*. (2020) model, with similar conclusions.

Finally, interpretation of the fatal incidence trajectories as proportional to the overall incidence trajectories rests on an assumption that the infection fatality rate is constant over time. There is evidence that the hospitalized case fatality rate declined in the 2 months or so after the peak of the first wave of infections (Dennis *et al*., 2021), with this effect not explicable by any detectable change in patient characteristics. However, on the ground changes in the severity threshold for admission would be very difficult to detect, seem likely at times when some hospitals were at or near capacity, and could also contribute to such a pattern. The Supporting Information includes a check of the impact that the reported improvements would have on the shape of inferred overall incidence. The peak incidence could be shifted by as much as a day later, and there would be a somewhat slower decline in incidence relative to the results plotted in Figure 2.

## 7 DISCUSSION

This paper does not prove that the peak in fatal infections in the United Kingdom preceded the first full lockdown by several days. Indeed, the failure to undertake the sampling that could have gathered data to directly measure infections early in the epidemic means that it will never be possible to be certain about timings then, given the substantial biases in clinical data other than deaths and fatal disease duration. What the results show is that, in the absence of strong assumptions, the currently most reliable openly available data strongly suggest that the decline in infections in the United Kingdom began before the first full lockdown, suggesting that the measures preceding lockdown may have been sufficient to bring the epidemic under control, and that community infections, unlike deaths, were probably at a low level well before the first lockdown was eased. Such a scenario would be consistent with the infection profile in Sweden, which began its decline in fatal infections shortly after the United Kingdom, but did so on the basis of measures well short of full lockdown.

The analysis does not in itself say what would have happened without full lockdown, and must obviously be weighed against other evidence. No currently available analysis will conclusively determine what would have happened without full lockdown, and the state of the art in causal inference is obviously a very long way from being able to answer this question. Models based on approximations to the mechanisms of epidemic transmission do not allow reliable answers to these causal questions either. This is particularly so given the paucity of data with which to validate their component assumptions – a paucity that only grows more acute as more detail is included in the models. These are not weather or climate models, based on the bulk properties of enormous numbers of physically well‐understood interactions of simple molecules, tested and refined against huge quantities of carefully measured calibration data collected worldwide over decades. Rather they are best working approximations constructed by experts given the limited information that could be rapidly assembled in a matter of months, and subject to all the uncertainty this implies. A model does not become a valid basis for casual inference merely by being described as mechanistic. As the above reanalysis using the Flaxman model serves to emphasize: the inclusion of model structure aiming to represent mechanism is no guarantee of improved statistical inference, and certainly not a justification for treating inference with mechanism‐based models as causal.

In the time since this work was first undertaken, other low assumption analyses have appeared, in particular looking for the coincidence of NPI introductions and changepoints in incidence, for example, in Germany and Spain. The results of this paper are in some alignment with such analyses for Germany (Wieland, 2020; Küchenhoff *et al*., 2021), which also suggest that a decline in incidence preceded the first full lockdown. Both are based on case data, which are problematic even in Germany which had mass (but not randomized) testing in place from the start of the epidemic. However, it seems likely that the biases in case data would lead to the start of decline in incidence being estimated as later than it really was, rather than earlier, so the qualitative conclusion is likely to be robust. In Spain, Santamaría and Hortal (2020) also identify substantial changes in rate of change of incidence before Spanish lockdown based on death series, but not sufficient to suggest a decline in incidence before lockdown. Based on pre‐print versions of the current paper, a number of researchers have also attempted to employ the basic idea of dynamic model‐free inference about incidence profiles, but via a simplified method. This method tries to impute date of infection by subtracting a random draw from the fatal duration distribution from each deceased patient's death date. This process is replicated to obtain an expected incidence profile. The method is invalid, as duration of disease is not independent of time of death, and it will tend to incorrectly show much less steep, or no, decline before lockdown. See the Supporting Information for a full discussion.

The results of applying the method to data up to mid‐February 2021 provide a picture rather consistent with the results for the first lockdown. In particular, the results preceding the first lockdown appear consistent with how the epidemic progressed under later restrictions short of lockdown. This is not the case for the published analyses suggesting high *R* and surging incidence on the eve of the first lockdown. The fact that school re‐opening does not appear to be followed by an increase in *R* is interesting: whether it relates to people deciding to keep school children apart from the vulnerable, which is anecdotally plausible, or to other factors, is unclear. While tempting, it is difficult to interpret the later patterns in terms of the new, apparently more infectious, variant that emerged in late 2020: there is confounding with seasonality of transmission, behavioural changes around the end of year holidays and with the roll out of effective vaccines from late December onwards. Greater clarity on these issues may emerge in future, particularly if the UK ONS Covid surveillance data eventually becomes public in raw form.

## Supporting information

The Web Appendix referenced in Sections 1,2,5,6, and 7 is available with this paper at the Biometrics website on Wiley Online Library, along with the R code and data for replicating the results.

## Data Availability

The data that support the findings in this paper are available in the Supporting Information of this article.
