# Supplementary material for: Inferring UK COVID‐19 fatal infection trajectories from daily mortality data: Were infections already in decline before the UK lockdowns?
Source: Biometrics. 2021 Apr 7:10.1111/biom.13462. Online ahead of print. doi: 10.1111/biom.13462 (PMC8251436; doi:10.1111/biom.13462)
Supplement: Supplementary file 1 — The Web Appendix referenced in Sections 1,2,5,6, and 7 is available with this paper at the Biometrics website on Wiley Online Library, along with the R code and data for replicating the results. [file BIOM-9999-0-s001.zip › supplement-biom.pdf]

Supporting Information for 'Inferring UK COVID-19 fatal infection trajectories from daily mortality data' by Simon N. Wood

March 19, 2021

Contents

1 Feasible direct inference of incidence from randomized PCR testing **1**

2 How not to infer fatal incidence **2**

3 Fatal disease duration distribution **3**

4 Possible age structure effects **5**

5 Further model checking of relaxed Flaxman model **6**

6 Sensitivity to mortality rate reductions **7**

1 Feasible direct inference of incidence from randomized PCR testing

Useful estimates of incidence can be obtained from properly randomized PCR surveillance testing, even using numbers of tests well within the laboratory capacity available early in the epidemic. This section provides a simple illustration of this, by sketching a method and showing its ability to capture incidence profiles at the sort of levels that are important for decision making - i.e. at a level slightly over 1 per

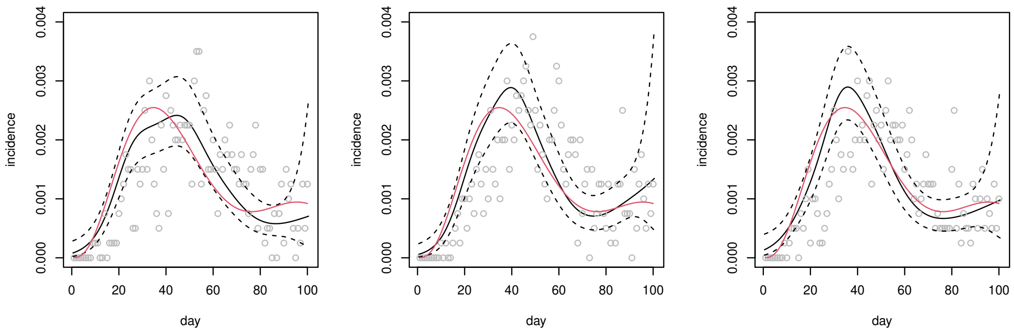

Figure 1: Three replicates of incidence rates reconstructed from simulated PCR testing data. True incidence is in red. Reconstructions and 2 standard error bands in black. Grey circles show the number of positive tests each day, divided by 4000. Obviously positivity lags incidence.

1000 per day. For illustrative purposes I consider a very simple model of PCR positivity in which the proportion, P, of people potentially testing positive is governed by the simple ODE model ($\dot{x}$ denoting the time derivative of x)

$$\dot{P} = f(t) - \delta P$$

where f is the incidence (strictly speaking of potential PCR positivity) as a proportion of the population, and $1/\delta$ is the mean duration of positivity. One could of course substitute any number of alternative models for the assumption of an exponential distribution of the time that subjects are PCR positive, without changing the basic approach discussed here. With only slightly more effort a stochastic formulation could also be substituted (although is likely to add little, given the large numbers involved). The number testing positive in random samples of size N from the population is then given by

$$y_i \sim \text{binom}(N, \alpha P)$$

where α is the test sensitivity (which is measurable in a reasonably direct manner). As in the main paper, we can represent f semi-parametrically, e.g. using a smoothing spline, so that

$$f(t) = \exp(\mathbf{X}_t \boldsymbol{\beta})$$

where $\mathbf{X}_t$ is a row vector of spline basis functions evaluated at time t. Writing the derivative of P w.r.t. β_j as P_{β_j} we have an ODE

$$\dot{P}_{\beta_j} = f(t) X_{tj} - \delta P_{\beta_j}$$

for each such derivative (known as *sensitivities* in this context). Given any value of $\boldsymbol{\beta}$ it is straightforward to solve for P and the sensitivities, for example by 4th order Runge-Kutta integration. Hence the log likelihood and its derivative are readily evaluated, and the empirical Bayes approach given in the main paper can be used to find the posterior models, $\hat{\boldsymbol{\beta}}$, an appropriate smoothing parameter and the large sample posterior covariance matrix. To avoid requiring the second derivative ODE system, $\hat{\boldsymbol{\beta}}$ can be obtained by quasi-Newton optimization, with the Hessian required for smoothing parameter update obtained from the first derivative of the log likelihood by finite differencing.

By way of illustration, data were simulated from such a model for 100 days, with 400 tests per day (2800 per week) conducted on randomly selected people from a general population subject to the true incidence curve shown in red in figure 1, and $\delta = 0.1$. The method was then used to reconstruct the incidence curve (here 100% sensitivity was assumed, since sensitivity is a simple scale parameter in this problem). Three random replicate reconstructions are shown in figure 1. Uncertainty is wide at the end of the data, but usable for 10 days earlier. Of course the swab to testing lag adds to this. Larger sample sizes would be needed if local/regional estimates are required, but for the 'whole country' picture considered in the main paper such direct estimation is clearly feasible.

2 How not to infer fatal incidence

Several researchers picked up the pre-print version of this paper (Wood, 2020) and have attempted to use the basic idea of inferring fatal incidence directly from death trajectories and the fatal disease distribution, but via a simple 'imputation' method. Suppose the ith patient died on day t_i. A random draw from the fatal disease duration distribution, τ_i, is subtracted from their death day to give an imputed infection day, $t_i - \tau_i$. Repeating this for all deaths generates an imputed fatal incidence curve. Repeating the imputation many times allows an expected incidence curve to be generated.

This method is not valid. It is completely plausible that duration of disease is independent of time of infection, but not of time of death. Further, unless incidence and deaths are at some constant equilibrium, duration of disease can not be independent of both time of infection *and* time of death: when deaths are

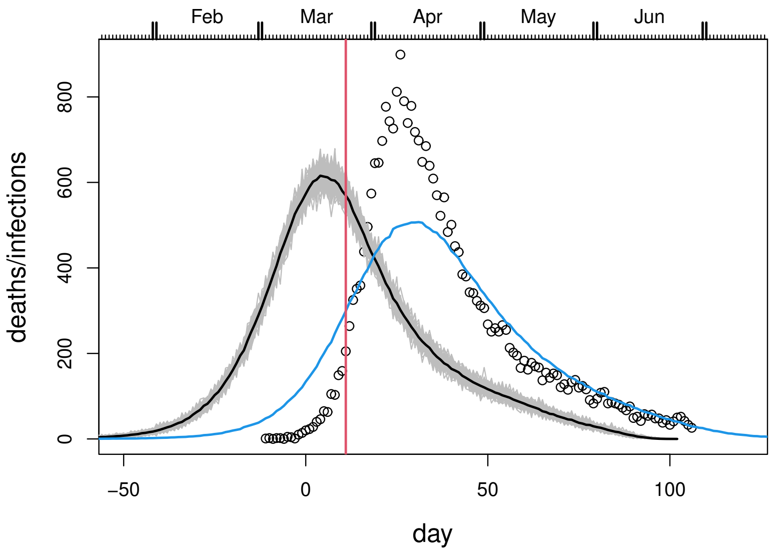

Figure 2: Illustration of the failure of a simple imputation method to correctly reconstruct an incidence curve consistent with the observed deaths. Symbols are observed English hospital deaths. Grey curves are infections imputed by the incorrect method given in the text. The black curve is the mean imputed incidence. The blue curve is the expected daily death trajectory implied by the black curve. If the method were correct it should pass through the data points. The red vertical line marks the first day of the first UK lockdown.

rising, we inevitably see the deaths from short duration diseases before those from longer durations. Since the imputation method assumes independence of t_i and τ_i it can not be valid. Figure 2 shows that this is not a minor concern. It shows incidences reconstructed using the described imputation method. I then added random draws from the fatal duration distribution to the imputed days of infection, to obtain the expected daily deaths implied by the imputed incidence trajectory (essentially the 'sanity check' applied in the main paper). The expected daily deaths are an exceedingly poor fit to the data.

3 Fatal disease duration distribution

Fatal disease duration data for England are available in the CHESS[1] database, access to which is restricted to particular research groups under strict conditions. With the kind help of Robert Verity from Imperial College I was able to access information on the distribution of fatal disease durations for 3274 deaths that occurred before 10 June 2020 with recorded symptom onset before 1 May. The information provided was a bar chart of the duration distribution by day, on condition that only the information about the model fitted to the data be distributed further. The data were not filtered to remove hospital acquired infections, but it was not possible to obtain data only for those with onset before hospitalization. This is problematic for two reasons. Firstly, for inferring the time course of community acquired fatal infections it is the distribution of fatal disease durations for community acquired infections that is required, which the raw data do not provide: for example, they contain substantial proportions of durations of 1-3 days that appear clinically implausible for deaths from community acquired COVID-19 (see, e.g. Huang et al., 2020; Wang et al., 2020; Zhou et al., 2020; Tay et al., 2020). Secondly the raw data are from a relatively small proportion of the total deaths. It is very unlikely that the ratio of hospital to community acquired infections in this sample is representative: for hospital acquired infections the onset of symptoms is presumably almost always known, and hence more likely to be recorded than for community acquired

[1]COVID-19 Hospitalisations in England Surveillance System

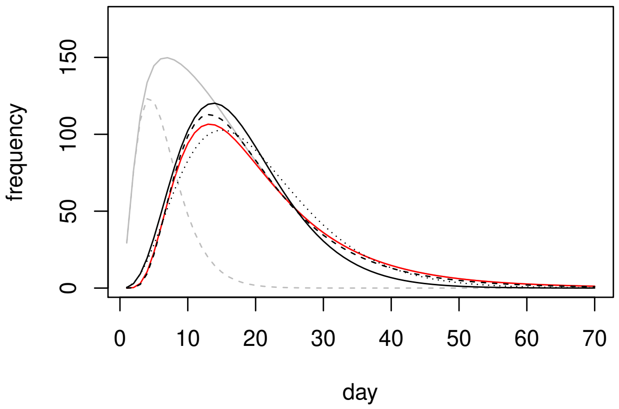

Figure 3: Onset to death duration distribution models. The red curve is the log-normal mixture component for community acquired infection fitted to the CHESS data, the dashed grey curve is the gamma mixture component representing hospital acquired infection and the continuous grey curve the combined model. The combined model is not directly usable: see text. The black curves are: continuous Verity et al. (2020); dashed Linton et al. (2020); dotted Wu et al. (2020). The mixture model was estimated by maximum likelihood, with the hospital acquired mixture proportion reduced until the profiled log likelihood was reduced to 4 below the MLE, to obtain the shortest mean community acquired duration consistent with the data under this model. The black curves in no way inform the red curve in the fitting.

infections. This makes the raw distribution unrepresentative of the distribution for all deaths and also not usefully informative about the proportion of all deaths that are from hospital acquired infection. Note also that without more extensive data access it is not possible to rule out that some proportion of what appear to be hospital acquired infections really represent data problems (for example recording onset day as hospital admission day).

To deal with these issues a two component mixture model was fitted to data digitized from the bar chart, consisting of a gamma distribution (representing hospital acquired infections) and a log-normal distribution (representing community acquired infections). Parameterization was such that the log-normal had the longer mean duration. The higher the gamma mixture proportion the larger the log-normal mean. To find the shortest mean community acquired duration defensible from the data, the gamma mixture proportion was reduced to the point at which the log likelihood was about 4 below the MLE (decreasing further decreases the log-likelihood sharply, pushes a χ^2 goodness of fit statistic into the significant range, and starts to suggest rather high probabilities of very short disease durations for the log-normal mixture component). This point has about 0.7 of the mixture contributed by the community infection component. The resulting log-normal community infection fit has a mean of 21 days and a standard deviation of 12.7. Longer durations would be slightly more consistent with the data under the mixture model, but given the aims of this paper it is better to use conservatively short estimates here. Figure 3 shows the various estimated distributions over the duration range observed in the CHESS data. The log-normal model has an earlier mode, but longer tail, than the Verity et al. (2020) model used in earlier versions of this paper.

It should perhaps be noted that this model was obtained before I was aware of Linton et al. (2020) and Wu et al. (2020). Note also that the data for this model were obtained before the decision to attribute deaths to Covid-19 only if there was a positive test within the 28 days preceding death: this may be the reason for the model's slightly heavier tail. Otherwise the results are broadly in agreement with those from the published studies.

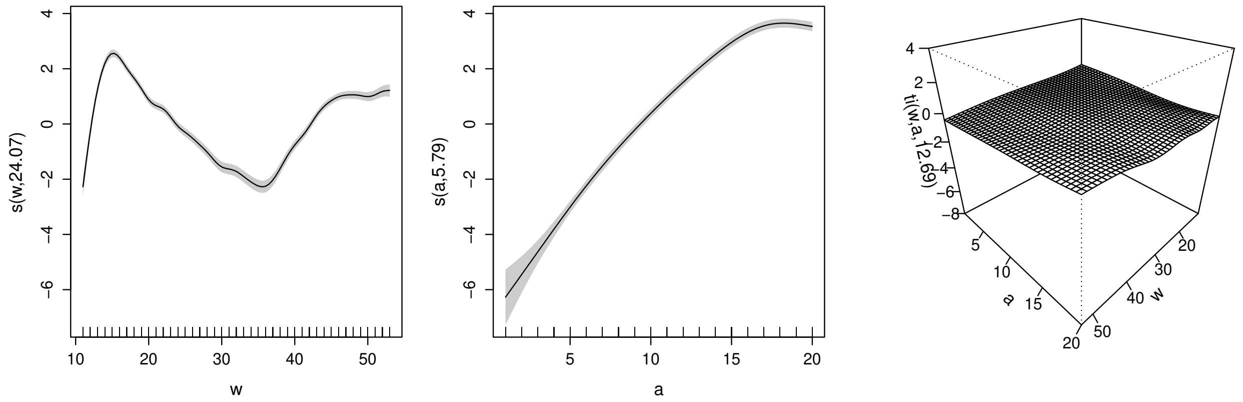

Figure 4: Generalized additive model term estimates on the log link scale, from fitting to England and Wales hospital death by age data. Left is the effect of week, middle of age group and right of their interaction. The interaction ranges over approximately -0.2 to 0.2, and is clearly a very small effect relative to the others.

Assuming independence of incubation period and onset to death period, the preceding fit and the McAloon et al. (2020) incubation period imply that the infection-to-death distribution for the community acquired infection component can be well modelled by a log-normal distribution with log scale mean and standard deviation of 3.19 and 0.44, respectively. That is a mean of 26.8 days and standard deviation of 12.4 days. The community infection distribution component is shown in blue in figure 2 of the paper.

More recently results of Robert Verity's own more detailed analysis of the CHESS data have appeared in Knock et al. (2020). The full fitted distribution is not given, but the figures that are reported imply a slightly shorter mean duration of just over 24 days. This is just under a day and a half less than for the mean duration for the average distribution used in the main paper, and within the uncertainty range considered in the paper.

4 Possible age structure effects

One possible concern is that if the distribution of fatal disease duration is strongly age dependent and the age distribution shifts over time, then the results of the paper's analysis could be biased in ways that could be difficult to correct. In fact Dennis et al. (2021) looked for temporal changes in patient characteristics including age as possible explanations for the mortality improvements that they report in the early months of the epidemic, but did not find age distribution changes in hospitalized patients. Additionally Knock et al. (2020) analysed English hospital data to parameterize a detailed age structured epidemic and hospital model, but while they report age effects on rates of hospitalization and transfer to ICU, with different distributions of time to death for ICU and general ward patients, those distributions are not reported to be age dependent.

While both the cited analyses rely on confidential data with stringently controlled access, it is possible to look for evidence of age distribution shifts in the weekly England and Wales Covid-19 deaths by age data publicly available from the UK Office for National Statistics[2]. These data give total England and Wales Covid-19 deaths each week in 20 age bands, <1, 1-4, 5-9 , ..., 85-89 and 90+. They also record the total number of Covid-19 deaths each week in care homes for the elderly in England and Wales. To look for age distribution changes in hospitalized patients, it is necessary to remove the care home deaths from the weekly totals. The care home deaths are not broken down by age, so I simply reduced the total deaths in the last three age classes by the same proportion, in order to reduce the each weekly total deaths

[2] https://www.ons.gov.uk/peoplepopulationandcommunity/birthsdeathsandmarriages/deaths/datasets/weeklyprovisionalfiguresondeathsregisteredinenglandandwales

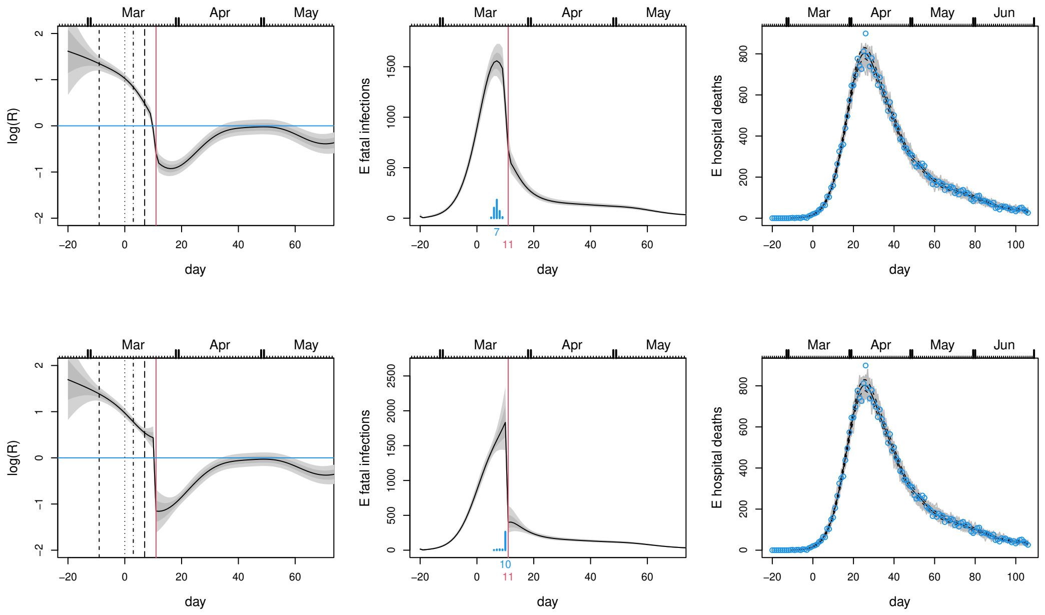

Figure 5: Model checking plots for the Flaxman et al. (2020) model. The upper row shows the results of applying a time dilation around lockdown to ensure that any very rapid change in R at that point can be accommodated by the model. The results are similar to the undilated case. The lower row shows a model which forces a step change at lockdown- notice the severe boundary uncertainty in the vicinity of lockdown introduced by this (see text for discussion). Even with this model R is about 1.5, substantially below the Flaxman et al. (2020) estimates of around 3 on the eve of lockdown.

by the correct amount.

A negative binomial generalized additive model was then fitted to the data, with the structure

$$\log\{E(\texttt{death}_i)\} = \alpha + f_1(a_i) + f_2(w_i) + f_3(a_i, w_i)$$

where a_i denotes the age class (a number from 1 to 20) and w_i is the week. f_1 and f_2 are univariate splines, while f_3 is a tensor product interaction spline, (without the main effects). Thus f_3 represents any change in age distribution of deaths over time. See Wood (2017) section 5.6.3 for details. f_3 is statistically significant, but the effect size is too small to be biologically significant. Figure 4 shows the estimated model components. Leaving the care home deaths in the totals leads to a slightly stronger interaction ranging from about -0.6 to 0.4 in the early weeks. This reflects the somewhat different dynamics of the care home epidemic relative to the community epidemic, as discussed in the main paper. The main effects are essentially unchanged from those shown.

5 Further model checking of relaxed Flaxman model

The time dilation check from the *Model checking* section of the paper was also applied to the relaxed Flaxman et al. (2020) model, with the results shown in the upper panel of figure 5. Again the results are qualitatively similar to the undilated case, despite modifying the model to favour sharp change in R at lockdown. Although highly problematic for the reasons discussed in the paper, the results of a check using a model in which a step change was forced to occur at lockdown is also shown in the lower row of figure 5. The boundary condition artefacts that this introduces are clearly visible, but in any case the inferred R on the eve of lockdown is about 1.5. This is substantially below the Flaxman et al. estimates of close to 3.

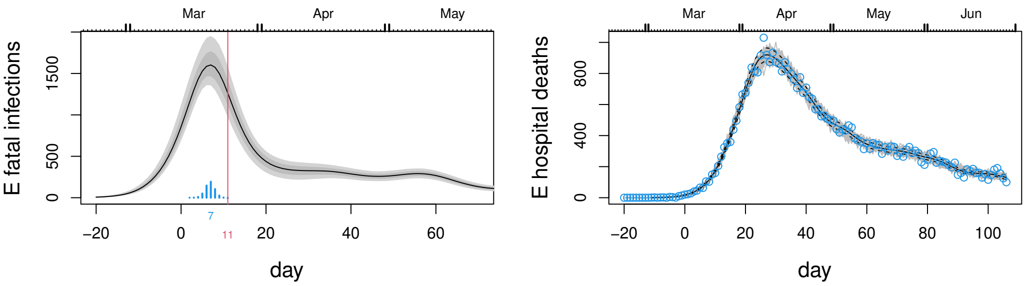

Figure 6: Sensitivity of the results to improvements in the IFR. To interpret the fatal incidence trajectories as proportional to overall disease incidence, the IFR has to be constant. There is evidence for this not being the case as hospital care has improved. These plots show inferred incidence from death data 'corrected' for the mortality rate improvements estimated in Dennis et al. (2021). Note the very slight rightward shift in the peak timing distribution, and somewhat slower decay in the incidence profile.

6 Sensitivity to mortality rate reductions

There is evidence for reductions in the hospital mortality rates in England from the week of 29th March 2020 until the end of June, with this reduction apparently not being attributable to any change in patient characteristics: Dennis et al. (2021) report mortality rates reducing by a multiplicative factor of about .985 per day (before then, if anything the death rates were increasing). While this does not undermine inference of fatal infection incidence, it obviously means that fatal disease incidence should probably not be interpreted as proportional to overall incidence. Given the uncertainties in the Dennis et al. (2021) results, a direct correction is difficult. Furthermore ruling out changes in severity of disease required for admission over the first wave is also not possible: for example, general practitioners (family doctors) were initially working with central guidance on when patients should self isolate, but not when they should be sent to hospital, so it seems unlikely that on the ground admission criteria were constant, especially at times when some hospitals were at or near capacity. However a sensitivity test is straightforward. The observed deaths each day can simply be scaled up by the ratio of the number of deaths expected without improvements to the number expected with improvements (assuming .985 per day improvement from 29th March). This has the effect of making the downward tail of the adjusted deaths series decay more slowly than for the observed deaths (see right panel of fig 6). Applying the method to the English hospital data then gives the results in figure 6. There is a shift in the inferred peak incidence to later, and the incidence decays more slowly, relative to the results shown in the main paper. Note that the mortality improvements only apply to hospital deaths, not care home deaths.

Acknowledgements

I am very grateful to Robert Verity for his unsolicited offer to help get access to the duration data for England, and for making the effort to get the permissions and follow through on this.

References

Dennis, J. M., A. P. McGovern, S. J. Vollmer, and B. A. Mateen (2021). Improving survival of critical care patients with coronavirus disease 2019 in england: a national cohort study, March to June 2020. *Critical care medicine 49*(2), 209.

Flaxman, S., S. Mishra, A. Gandy, H. J. T. Unwin, T. A. Mellan, H. Coupland, C. Whittaker, H. Zhu, T. Berah, J. W. Eaton, et al. (2020). Estimating the effects of non-pharmaceutical interventions on COVID-19 in Europe. *Nature 584*(7820), 257–261.

Huang, C., Y. Wang, X. Li, L. Ren, J. Zhao, Y. Hu, L. Zhang, G. Fan, J. Xu, X. Gu, et al. (2020). Clinical features of patients infected with 2019 novel coronavirus in Wuhan, China. *The Lancet 395*(10223), 497–506.

Knock, E. S., L. K. Whittles, J. A. Lees, P. N. Perez Guzman, R. Verity, R. G. Fitzjohn, K. A. M. Gaythorpe, N. Imai, W. Hinsley, L. C. Okell, A. Rosello, N. Kantas, C. E. Walters, S. Bhatia, O. J. Watson, C. Whittaker, L. Cattarino, A. Boonyasiri, B. A. Djaafara, K. Fraser, H. Fu, H. Wang, X. Xi, C. A. Donnelly, E. Jauneijaite, D. J. Laydon, P. J. White, A. C. Ghani, N. M. Ferguson, A. Cori, and M. Baguelin (2020). Report 41: The 2020 SARS-CoV-2 epidemic in England: key epidemiological drivers and impact of interventions. *Imperial College London*.

Linton, N. M., T. Kobayashi, Y. Yang, K. Hayashi, A. R. Akhmetzhanov, S.-m. Jung, B. Yuan, R. Kinoshita, and H. Nishiura (2020). Incubation period and other epidemiological characteristics of 2019 novel coronavirus infections with right truncation: a statistical analysis of publicly available case data. *Journal of clinical medicine 9*(2), 538.

McAloon, C., Á. Collins, K. Hunt, A. Barber, A. W. Byrne, F. Butler, M. Casey, J. Griffin, E. Lane, D. McEvoy, et al. (2020). Incubation period of COVID-19: a rapid systematic review and meta-analysis of observational research. *BMJ open 10*(8), e039652.

Tay, M. Z., C. M. Poh, L. Rénia, P. A. MacAry, and L. F. Ng (2020). The trinity of COVID-19: immunity, inflammation and intervention. *Nature Reviews Immunology*, 1–12.

Verity, R., L. C. Okell, I. Dorigatti, P. Winskill, C. Whittaker, N. Imai, G. Cuomo-Dannenburg, H. Thompson, P. G. Walker, H. Fu, et al. (2020). Estimates of the severity of coronavirus disease 2019: a model-based analysis. *The Lancet Infectious Diseases*.

Wang, D., B. Hu, C. Hu, F. Zhu, X. Liu, J. Zhang, B. Wang, H. Xiang, Z. Cheng, Y. Xiong, et al. (2020). Clinical characteristics of 138 hospitalized patients with 2019 novel coronavirus–infected pneumonia in Wuhan, China. *Jama 323*(11), 1061–1069.

Wood, S. N. (2017). *Generalized Additive Models: An Introduction with R* (2 ed.). Boca Raton, FL: CRC press.

Wood, S. N. (2020). Did COVID-19 infections decline before UK lockdown? *arXiv preprint ArXiv:2005.02090*.

Wu, J. T., K. Leung, M. Bushman, N. Kishore, R. Niehus, P. M. de Salazar, B. J. Cowling, M. Lipsitch, and G. M. Leung (2020). Estimating clinical severity of COVID-19 from the transmission dynamics in Wuhan, China. *Nature Medicine 26*(4), 506–510.

Zhou, F., T. Yu, R. Du, G. Fan, Y. Liu, Z. Liu, J. Xiang, Y. Wang, B. Song, X. Gu, et al. (2020). Clinical course and risk factors for mortality of adult inpatients with COVID-19 in Wuhan, China: a retrospective cohort study. *The Lancet 395*(10229), 1054–1062.
